# Supplementary material for: Cytochrome P450 Surface Domains Prevent the β-Carotene Monohydroxylase CYP97H1 of Euglena gracilis from Acting as a Dihydroxylase
Source: Biomolecules. 2023 Feb 15;13(2):366. doi: 10.3390/biom13020366 (PMC9953315; doi:10.3390/biom13020366)
Supplement: Supplementary file 1 [file biomolecules-13-00366-s001.zip › biomolecules-2114670-supplementary.pdf]

Supplementary Information

Cytochrome P450 surface domains prevent the β-carotene mon-ohydroxylase CYP97H1 of *Euglena gracilis* from acting as a di-hydroxylase.

Thomas Lautier[1,2,3*]; Derek J. Smith[1]; Lay Kien Yang[1]; Xixian Chen[1]; Congqiang Zhang[1]; Gilles Truan[2]; Nic D Lindley[1,2]

1 Singapore Institute of Food and Biotechnology Innovation (SIFBI), Agency for Science, Technology and Research (A*STAR), Singapore 138669, Singapore.
2 Toulouse Biotechnolgy Institute, Université de Toulouse, CNRS, INRAE, INSA, 31077 Toulouse, France.
3 CNRS@CREATE, 1 Create Way, #08-01 Create Tower, Singapore 138602.
* Correspondence: thomas.lautier@cnrs.fr; Tel.: +33(0)567048813

Table S1. Protein sequences of the redox partner and the cytochrome P450 variants, truncated forms and the chimeras. The *E. coli* codon optimized sequences were cloned on the corresponding plasmid pTLXX.

**FER1 Ferredoxin *S. oleracea***
MAAYKVTLVTPTGNVEFQCPDDVYILDAAEEEGIDLPYSCRAGSCSSCAGKLKTGSLNQDDQSFLDDDQIDEGWVL<br>TCAAYPVSDVTIETHKEEELTA*
**Ferredoxin reductase *S. oleracea***
MQIASDVEAPPPAPAKVEKHSKKMEEGITVNKFKPKTPYVGRCLLNTKITGDDAPGETWHMVFSHEGEIPYREGQS<br>VGVIPDGEDKNGKPHKLRLYSIASSALGDFGDAKSVSLCVKRLIYTNDAGETIKGVCSNFLCDLKPGAEVKLTGPVG<br>KEMLMPKDPNATIIMLGTGTGIAPFRSFLWKMFFEKHDDYKFNGLAWLFLGVPTSSSLLYKEEFEKMKEKAPDNFRL<br>DFAVSREQTNEKGEKMYIQTRMAQYAVELWEMLKKDNTYFYMCGLKGMEKGIDDIMVSLAAAEGIDWIEYKRQL<br>KKAEQWNVEVY*
**pTL45: bov-CYP97H1**
MALLLAVFLGLSCLLLLSLWGSRRKTPNRSQCLLAFTGEREGLRHQELVPIRNLDINCHGVKTSCTQLQATVEPSPEQ<br>ESQLPRAEDMAIGVTAQEYVTHYIQNVAQFFVPMKWEDNIPVVSVFDIFKWGAVDIPMERLLQSKLTDVFTGGFQDI<br>TGVPVFILLHRYMALSPIYKLCIGPRSVVVISDAVAVKHILRSEVGKYDKGILAEVLKPIMGKGLIPADTITWLTRRRQL<br>KPAFHQKWLHDQLTLYSTVGNRLVAFLAARPGQTIDMQERFCSASLDIIGKAVFNYEFGSITRESPVIQAVYAVMRE<br>AERRASSIVPYWQLPGGTREFDQHMKVLDDVLTSLVEQCVQQVSTEEDEEPQKGNNSLLRFLVEARGQDVTNQQL<br>RDDLMTMLIAGHETTAATLTWALHELTKPENRDFLKRVKAEVDSVLGLRDFITLDDVKQMPLVRYSLVEALRLYPA<br>PPMLIRRCLKEDHLTGVGPFSAGMTIKPGQDVMLATWSLNRDQRLWGPDADKYNPLRFYTAVHGSPEYKAAGWA<br>GFDPARVRGLYPDENAADFGFIPFGGGGRKCMGDQFAILESSVLLSMLLRDFSFEAADTVTLGMGATIFAKEGLMM<br>KVTARPPQPPDQDEASPVAVASDALLSSVA*
**pTL68: full-length CYP97H1**
MDGDSIACTPFLRAWHWHHGLILVASSMLTIVIINAQAQQLTISSPLKSSTRSTTSVVLGGTQRYRLPPTAGTAVGTV<br>VAHPNNANPSKARLHGMLVNKAIFLPLLLPIVAGIAWLRRKTPNRSQCLLAFTGEREGLRHQELVPIRNLDINCHG<br>VKTSCTQLQATVEPSPEQESQLPRAEDMAIGVTAQEYVTHYIQNVAQFFVPMKWEDNIPVVSVFDIFKWGAVDIPM<br>ERLLQSKLTDVFTGGFQDITGVPVFILLHRYMALSPIYKLCIGPRSVVVISDAVAVKHILRSEVGKYDKGILAEVLKPIM<br>GKGLIPADTITWLTRRRQLKPAFHQKWLHDQLTLYSTVGNRLVAFLAARPGQTIDMQERFCSASLDIIGKAVFNYEF<br>GSITRESPVIQAVYAVMREAERRASSIVPYWQLPGGTREFDQHMKVLDDVLTSLVEQCVQQVSTEEDEEPQKGNNSL<br>LRFLVEARGQDVTNQQLRDDLMTMLIAGHETTAATLTWALHELTKPENRDFLKRVKAEVDSVLGLRDFITLDDVK<br>QMPLVRYSLVEALRLYPAPPMLIRRCLKEDHLTGVGPFSAGMTIKPGQDVMLATWSLNRDQRLWGPDADKYNPLR<br>FYTAVHGSPEYKAAGWAGFDPARVRGLYPDENAADFGFIPFGGGGRKCMGDQFAILESSVLLSMLLRDFSFEAADT<br>VTLGMGATIFAKEGLMMKVTARPPQPPDQDEASPVAVASDALLSSVA*
**pTL70: bov-capless-CYP97H1**

MALLLAVFLGLSCLLLLSLWGSSPIYKLCIGPRSVVVISDAVAVKHILRSEVGKYDKGILAEVLKPIMGKGLIPADTITW
LTRRRQLKPAFHQKWLHDQLTLYSTVGNRLVAFLAARPGQTIDMQERFCSASLDIIGKAVFNYEFGSITRESPVIQAV
YAVMREAERRASSIVPYWQLPGGTREFDQHMKVLDDVLTSLVEQCVQQVSTEEDEEPQKGNNSLLRFLVEARGQD
VTNQQLRDDLMTMLIAGHETTAATLTWALHELTKPENRDFLKRVKAEVDSVLGLRDFITLDDVKQMPLVRYSLVE
ALRLYPAPPMLIRRCLKEDHLTGVGPFSAGMTIKPGQDVMLATWSLNRDQRLWGPDADKYNPLRFYTAVHGSPEY
KAAGWAGFDPARVRGLYPDENAADFGFIPFGGGGRKCMGDQFAILESSVLLSMLLRDFSFEAADTVTLGMGATIFA
KEGLMMKVTARPPQPPDQDEASPVAVASDALLSSVA*

pTL71: supertruncated-CYP97H1

MSPIYKLCIGPRSVVVISDAVAVKHILRSEVGKYDKGILAEVLKPIMGKGLIPADTITWLTRRRQLKPAFHQKWLHDQ
LTLYSTVGNRLVAFLAARPGQTIDMQERFCSASLDIIGKAVFNYEFGSITRESPVIQAVYAVMREAERRASSIVPYWQL
PGGTREFDQHMKVLDDVLTSLVEQCVQQVSTEEDEEPQKGNNSLLRFLVEARGQDVTNQQLRDDLMTMLIAGHET
TAATLTWALHELTKPENRDFLKRVKAEVDSVLGLRDFITLDDVKQMPLVRYSLVEALRLYPAPPMLIRRCLKEDHLT
GVGPFSAGMTIKPGQDVMLATWSLNRDQRLWGPDADKYNPLRFYTAVHGSPEYKAAGWAGFDPARVRGLYPDE
NAADFGFIPFGGGGRKCMGDQFAILESSVLLSMLLRDFSFEAADTVTLGMGATIFAKEGLMMKVTARPPQPPDQDE
ASPVAVASDALLSSVA*

pTL72: tCYP97H1

MGSRRKTPNRSQCLLAFTGEREGLRHQELVPIRNLDINCHGVKTSCTQLQATVEPSPEQESQLPRAEDMAIGVTAQE
YVTHYIQNVAQFFVPMKWEDNIPVVSVFDIFKWGAVDIPMERLLQSKLTDVFTGGFQDITGVPVFILLHRYMALSPIY
KLCIGPRSVVVISDAVAVKHILRSEVGKYDKGILAEVLKPIMGKGLIPADTITWLTRRRQLKPAFHQKWLHDQLTLYS
TVGNRLVAFLAARPGQTIDMQERFCSASLDIIGKAVFNYEFGSITRESPVIQAVYAVMREAERRASSIVPYWQLPGGT
REFDQHMKVLDDVLTSLVEQCVQQVSTEEDEEPQKGNNSLLRFLVEARGQDVTNQQLRDDLMTMLIAGHETTAAT
LTWALHELTKPENRDFLKRVKAEVDSVLGLRDFITLDDVKQMPLVRYSLVEALRLYPAPPMLIRRCLKEDHLTGVGP
FSAGMTIKPGQDVMLATWSLNRDQRLWGPDADKYNPLRFYTAVHGSPEYKAAGWAGFDPARVRGLYPDENAAD
FGFIPFGGGGRKCMGDQFAILESSVLLSMLLRDFSFEAADTVTLGMGATIFAKEGLMMKVTARPPQPPDQDEASPVA
VASDALLSSVA*

pTL73: capless CYP97H1

MDGDSIACTPFLRAWHWHHGLILVASSMLTIVIINAQAQQLTISSPLKSSTRSTTSVVLGGTQRYRLPPTAGTAVGTV
VAHPNNANPSKARLHGMLVNKAIFLPLLLPIVAGIAWLSPIYKLCIGPRSVVVISDAVAVKHILRSEVGKYDKGILAE
VLKPIMGKGLIPADTITWLTRRRQLKPAFHQKWLHDQLTLYSTVGNRLVAFLAARPGQTIDMQERFCSASLDIIGKA
VFNYEFGSITRESPVIQAVYAVMREAERRASSIVPYWQLPGGTREFDQHMKVLDDVLTSLVEQCVQQVSTEEDEEPQ
KGNNSLLRFLVEARGQDVTNQQLRDDLMTMLIAGHETTAATLTWALHELTKPENRDFLKRVKAEVDSVLGLRDFI
TLDDVKQMPLVRYSLVEALRLYPAPPMLIRRCLKEDHLTGVGPFSAGMTIKPGQDVMLATWSLNRDQRLWGPDAD
KYNPLRFYTAVHGSPEYKAAGWAGFDPARVRGLYPDENAADFGFIPFGGGGRKCMGDQFAILESSVLLSMLLRDFS
FEAADTVTLGMGATIFAKEGLMMKVTARPPQPPDQDEASPVAVASDALLSSVA*

pTL79: M182-CYP97H1

MAIGVTAQEYVTHYIQNVAQFFVPMKWEDNIPVVSVFDIFKWGAVDIPMERLLQSKLTDVFTGGFQDITGVPVFILL
HRYMALSPIYKLCIGPRSVVVISDAVAVKHILRSEVGKYDKGILAEVLKPIMGKGLIPADTITWLTRRRQLKPAFHQK
WLHDQLTLYSTVGNRLVAFLAARPGQTIDMQERFCSASLDIIGKAVFNYEFGSITRESPVIQAVYAVMREAERRASSI
VPYWQLPGGTREFDQHMKVLDDVLTSLVEQCVQQVSTEEDEEPQKGNNSLLRFLVEARGQDVTNQQLRDDLMTM
LIAGHETTAATLTWALHELTKPENRDFLKRVKAEVDSVLGLRDFITLDDVKQMPLVRYSLVEALRLYPAPPMLIRRC
LKEDHLTGVGPFSAGMTIKPGQDVMLATWSLNRDQRLWGPDADKYNPLRFYTAVHGSPEYKAAGWAGFDPARV
RGLYPDENAADFGFIPFGGGGRKCMGDQFAILESSVLLSMLLRDFSFEAADTVTLGMGATIFAKEGLMMKVTARPP
QPPDQDEASPVAVASDALLSSVA*

pTL80: M206-CYP97H1

MKWEDNIPVVSVFDIFKWGAVDIPMERLLQSKLTDVFTGGFQDITGVPVFILLHRYMALSPIYKLCIGPRSVVVISDAV
AVKHILRSEVGKYDKGILAEVLKPIMGKGLIPADTITWLTRRRQLKPAFHQKWLHDQLTLYSTVGNRLVAFLAARPG
QTIDMQERFCSASLDIIGKAVFNYEFGSITRESPVIQAVYAVMREAERRASSIVPYWQLPGGTREFDQHMKVLDDVLT
SLVEQCVQQVSTEEDEEPQKGNNSLLRFLVEARGQDVTNQQLRDDLMTMLIAGHETTAATLTWALHELTKPENRD
FLKRVKAEVDSVLGLRDFITLDDVKQMPLVRYSLVEALRLYPAPPMLIRRCLKEDHLTGVGPFSAGMTIKPGQDVML
ATWSLNRDQRLWGPDADKYNPLRFYTAVHGSPEYKAAGWAGFDPARVRGLYPDENAADFGFIPFGGGGRKCMG
DQFAILESSVLLSMLLRDFSFEAADTVTLGMGATIFAKEGLMMKVTARPPQPPDQDEASPVAVASDALLSSVA*

pTL81: tCYP97H1-fullAt: entire F′-G′ loop 26 residues from CYP97A3At

MGSRRKTPNRSQCLLAFTGEREGLRHQELVPIRNLDINCHGVKTSCTQLQATVEPSPEQESQLPRAEDMAIGVTAQE YVTHYIQNVAQFFVPMKWEDNIPVVSVFDIFKWGAVDIPMERLLQSKLTDVFTGGFQDITGVPVFILLHRYMALSPIY KLCIGPRSVVVISDAVAVKHILRSEVGKYDKGILAEVLKPIMGKGLIPADTITWLTRRRQLKPAFHQKWLHDQLTLYS TVGNRLVAFLAARPGQTIDMQERFCSASLDIIGKAVFNYEFGSITRESPVIQAVYAVMREAERRSVSPIPVWDIPIWKD ISPRQRKVAQHMKVLDDVLTSLVEQCVQQVSTEEDEEPQKGNNSLLRFLVEARGQDVTNQQLRDDLMTMLIAGHE TTAATLTWALHELTKPENRDFLKRVKAEVDSVLGLRDFITLDDVKQMPLVRYSLVEALRLYPAPPMLIRRCLKEDHL TGVGPFSAGMTIKPGQDVMLATWSLNRDQRLWGPDADKYNPLRFYTAVHGSPEYKAAGWAGFDPARVRGLYPDE NAADFGFIPFGGGGRKCMGDQFAILESSVLLSMLLRDFSFEADTVTLGMGATIFAKEGLMMKVTARPPQPPDQDE ASPVAVASDALLSSVA*

pTL82: tCYP97H1-6At: 6 missing counterpart residues from CYP97A3At

MGSRRKTPNRSQCLLAFTGEREGLRHQELVPIRNLDINCHGVKTSCTQLQATVEPSPEQESQLPRAEDMAIGVTAQE YVTHYIQNVAQFFVPMKWEDNIPVVSVFDIFKWGAVDIPMERLLQSKLTDVFTGGFQDITGVPVFILLHRYMALSPIY KLCIGPRSVVVISDAVAVKHILRSEVGKYDKGILAEVLKPIMGKGLIPADTITWLTRRRQLKPAFHQKWLHDQLTLYS TVGNRLVAFLAARPGQTIDMQERFCSASLDIIGKAVFNYEFGSITRESPVIQAVYAVMREAERRASSIVPYWQLPGGK GYQSPSREFDQHMKVLDDVLTSLVEQCVQQVSTEEDEEPQKGNNSLLRFLVEARGQDVTNQQLRDDLMTMLIAGH ETTAATLTWALHELTKPENRDFLKRVKAEVDSVLGLRDFITLDDVKQMPLVRYSLVEALRLYPAPPMLIRRCLKEDH LTGVGPFSAGMTIKPGQDVMLATWSLNRDQRLWGPDADKYNPLRFYTAVHGSPEYKAAGWAGFDPARVRGLYPD ENAADFGFIPFGGGGRKCMGDQFAILESSVLLSMLLRDFSFEADTVTLGMGATIFAKEGLMMKVTARPPQPPDQD EASPVAVASDALLSSVA*

pTL83: tCYP97A3At

MSFPSTVKNGLSKIGIPSNVLDFMFDWTGSDQDYPKVPEAKGSIQAVRNEAFFIPLYELFLTYGGIFRLTFGPKSFLIVS DPSIAKHILKDNAKAYSKGILAEILDFVMGKGLIPADGEIWRRRRRAIVPALHQKYVAAMISLFGEASDRLCQKLDA AALKGEEVEMESLFSRLTLDIIGKAVFNYDFDSLTNDTGVIEAVYTVLREAEDRSVSPIPVWDIPIWKDISPRQRKVATS LKLINDTLDDLIATCKRMVEEEELQFHEEYMNERDPSILHFLLASGDDVSSKQLRDDLMTMLIAGHETSAAVLTWTF YLLTTEPSVVAKLQEEVDSVIGDRFPTIQDMKKLKYTTRVMNESLRLYPQPPVLIRRSIDNDILGEYPIKRGEDIFISVW NLHRSPLHWDDAEKFNPERWPLDGPNPNETNQNFSYLPFGGGPRKCIGDMFASFENVVAIAMLIRRFNFQIAPGAP PVKMTTGATIHTTEGLKLTVTKRTKPLDIPSVPILPMDTSRDEVSSALS*

pTL84: Nterm97H1-CYP97A3At

MGSRRKTPNRSQCLLAFTGEREGLRHQELVPIRNLDINCHGVKTSCTQLQATVEPSPEQESQLPRAEDMAIGVTAQE YVTHYIQNVAQFFVPMKWEDNIPVVSVFDIFKWGAVDIPMERLLQSKLTDVFTGGFQDITGVPVFILLHRYMALSFPS TVKNGLSKIGIPSNVLDFMFDWTGSDQDYPKVPEAKGSIQAVRNEAFFIPLYELFLTYGGIFRLTFGPKSFLIVSDPSIAK HILKDNAKAYSKGILAEILDFVMGKGLIPADGEIWRRRRRAIVPALHQKYVAAMISLFGEASDRLCQKLDAAALKGE EVEMESLFSRLTLDIIGKAVFNYDFDSLTNDTGVIEAVYTVLREAEDRSVSPIPVWDIPIWKDISPRQRKVATSLKLIND TLDDLIATCKRMVEEEELQFHEEYMNERDPSILHFLLASGDDVSSKQLRDDLMTMLIAGHETSAAVLTWTFYLLTTE PSVVAKLQEEVDSVIGDRFPTIQDMKKLKYTTRVMNESLRLYPQPPVLIRRSIDNDILGEYPIKRGEDIFISVWNLHRSP LHWDDAEKFNPERWPLDGPNPNETNQNFSYLPFGGGPRKCIGDMFASFENVVAIAMLIRRFNFQIAPGAPPVKMTT GATIHTTEGLKLTVTKRTKPLDIPSVPILPMDTSRDEVSSALS*

pTL88: tCYP97H1 with substrate channel from CYP97AAt

MGSRRKTPNRSQCLLAFTGEREGLRHQELVPIRNLDINCHGVKTSCTQLQATVEPSPEQESQLPRAEDMAIGVTAQE YVTHYIQNVAQFFVPMKWEDNIPVVSVFDIFKWGAVDIPMERLLQSKLTDVFTGGFQDITGVPFFILLHRYMALSPIY KLCFGPRSFVVISDAVAVKHILRSEVGKYDKGILAEILKPIMGKGLIPADTITWLTRRRQLKPAFHQKWLHDQLTLYS TVGNRLVAFLAARPGQTIDMQERFCSASLDIIGKAVFNYEFGSITRESPVIQAVYAVMREAERRASSIIPYWQLPGGTR EFDQHMKVLDDVLTSLVEQCVQQVSTEEDEEPQKGNNSLLRFLVEARGQDVTNQQLRDDLMTMLIAGHETTAATL TWALHELTKPENRDFLKRVKAEVDSVLGLRDFITLDDVKQMPLVRYSLVEALRLYPAPPVLIRRCLKEDHLTGVGPF SAGMTIKPGQDVFLATWSLNRDQRLWGPDADKYNPLRFYTAVHGSPEYKAAGWAGFDPARVRGLYPDENAADF GFIPFGGGGRKCMGDQFAILESSVLLSMLLRDFSFEADTVTLGMGATIFAKEGLMMKVTARPPQPPDQDEASPVAV ASDALLSSVA*

pTL91: tCYP97H1 F105A

MGSRRKTPNRSQCLLAFTGEREGLRHQELVPIRNLDINCHGVKTSCTQLQATVEPSPEQESQLPRAEDMAIGVTAQE YVTHYIQNVAQFFVPMKWEDNIPVVSVADIFKWGAVDIPMERLLQSKLTDVFTGGFQDITGVPVFILLHRYMALSPI YKLCIGPRSVVVISDAVAVKHILRSEVGKYDKGILAEVLKPIMGKGLIPADTITWLTRRRQLKPAFHQKWLHDQLTLY STVGNRLVAFLAARPGQTIDMQERFCSASLDIIGKAVFNYEFGSITRESPVIQAVYAVMREAERRASSIVPYWQLPGGT

REFDQHMKVLDDVLTSLVEQCVQQVSTEEDEEPQKGNNSLLRFLVEARGQDVTNQQLRDDLMTMLIAGHETTAAT
LTWALHELTKPENRDFLKRVKAEVDSVLGLRDFITLDDVKQMPLVRYSLVEALRLYPAPPMLIRRCLKEDHLTGVGP
FSAGMTIKPGQDVMLATWSLNRDQRLWGPDADKYNPLRFYTAVHGSPEYKAAGWAGFDPARVRGLYPDENAAD
FGFIPFGGGGRKCMGDQFAILESSVLLSMLLRDFSFEAADTVTLGMGATIFAKEGLMMKVTARPPQPPDQDEASPVA
VASDALLSSVA*

pTL92: tCYP97H1 F108A

MGSRRKTPNRSQCLLAFTGEREGLRHQELVPIRNLDINCHGVKTSCTQLQATVEPSPEQESQLPRAEDMAIGVTAQE
YVTHYIQNVAQFFVPMKWEDNIPVVSVFDIAKWGAVDIPMERLLQSKLTDVFTGGFQDITGVPVFILLHRYMALSPI
YKLCIGPRSVVVISDAVAVKHILRSEVGKYDKGILAEVLKPIMGKGLIPADTITWLTRRRQLKPAFHQKWLHDQLTLY
STVGNRLVAFLAARPGQTIDMQERFCSASLDIIGKAVFNYEFGSITRESPVIQAVYAVMREAERRASSIVPYWQLPGGT
REFDQHMKVLDDVLTSLVEQCVQQVSTEEDEEPQKGNNSLLRFLVEARGQDVTNQQLRDDLMTMLIAGHETTAAT
LTWALHELTKPENRDFLKRVKAEVDSVLGLRDFITLDDVKQMPLVRYSLVEALRLYPAPPMLIRRCLKEDHLTGVGP
FSAGMTIKPGQDVMLATWSLNRDQRLWGPDADKYNPLRFYTAVHGSPEYKAAGWAGFDPARVRGLYPDENAAD
FGFIPFGGGGRKCMGDQFAILESSVLLSMLLRDFSFEAADTVTLGMGATIFAKEGLMMKVTARPPQPPDQDEASPVA
VASDALLSSVA*

pTL93: tCYP97H1 F129A

MGSRRKTPNRSQCLLAFTGEREGLRHQELVPIRNLDINCHGVKTSCTQLQATVEPSPEQESQLPRAEDMAIGVTAQE
YVTHYIQNVAQFFVPMKWEDNIPVVSVFDIFKWGAVDIPMERLLQSKLTDVATGGFQDITGVPVFILLHRYMALSPI
YKLCIGPRSVVVISDAVAVKHILRSEVGKYDKGILAEVLKPIMGKGLIPADTITWLTRRRQLKPAFHQKWLHDQLTLY
STVGNRLVAFLAARPGQTIDMQERFCSASLDIIGKAVFNYEFGSITRESPVIQAVYAVMREAERRASSIVPYWQLPGGT
REFDQHMKVLDDVLTSLVEQCVQQVSTEEDEEPQKGNNSLLRFLVEARGQDVTNQQLRDDLMTMLIAGHETTAAT
LTWALHELTKPENRDFLKRVKAEVDSVLGLRDFITLDDVKQMPLVRYSLVEALRLYPAPPMLIRRCLKEDHLTGVGP
FSAGMTIKPGQDVMLATWSLNRDQRLWGPDADKYNPLRFYTAVHGSPEYKAAGWAGFDPARVRGLYPDENAAD
FGFIPFGGGGRKCMGDQFAILESSVLLSMLLRDFSFEAADTVTLGMGATIFAKEGLMMKVTARPPQPPDQDEASPVA
VASDALLSSVA*

pTL94: tCYP97H1 F133A

MGSRRKTPNRSQCLLAFTGEREGLRHQELVPIRNLDINCHGVKTSCTQLQATVEPSPEQESQLPRAEDMAIGVTAQE
YVTHYIQNVAQFFVPMKWEDNIPVVSVFDIFKWGAVDIPMERLLQSKLTDVFTGGAQDITGVPVFILLHRYMALSPI
YKLCIGPRSVVVISDAVAVKHILRSEVGKYDKGILAEVLKPIMGKGLIPADTITWLTRRRQLKPAFHQKWLHDQLTLY
STVGNRLVAFLAARPGQTIDMQERFCSASLDIIGKAVFNYEFGSITRESPVIQAVYAVMREAERRASSIVPYWQLPGGT
REFDQHMKVLDDVLTSLVEQCVQQVSTEEDEEPQKGNNSLLRFLVEARGQDVTNQQLRDDLMTMLIAGHETTAAT
LTWALHELTKPENRDFLKRVKAEVDSVLGLRDFITLDDVKQMPLVRYSLVEALRLYPAPPMLIRRCLKEDHLTGVGP
FSAGMTIKPGQDVMLATWSLNRDQRLWGPDADKYNPLRFYTAVHGSPEYKAAGWAGFDPARVRGLYPDENAAD
FGFIPFGGGGRKCMGDQFAILESSVLLSMLLRDFSFEAADTVTLGMGATIFAKEGLMMKVTARPPQPPDQDEASPVA
VASDALLSSVA*

pTL95: K117-A127At-CYP97H1

MGSRRKTPNRSQCLLAFTGEREGLRHQELVPIRNLDINCHGVKTSCTQLQATVEPSPEQESQLPRAEDMAIGVTAQE
YVTHYIQNVAQFFVPMKWEDNIPVVKGSIQAVRNEAVFILLHRYMALSPIYKLCIGPRSVVVISDAVAVKHILRSEVG
KYDKGILAEVLKPIMGKGLIPADTITWLTRRRQLKPAFHQKWLHDQLTLYSTVGNRLVAFLAARPGQTIDMQERFCS
ASLDIIGKAVFNYEFGSITRESPVIQAVYAVMREAERRASSIVPYWQLPGGTREFDQHMKVLDDVLTSLVEQCVQQVS
TEEDEEPQKGNNSLLRFLVEARGQDVTNQQLRDDLMTMLIAGHETTAATLTWALHELTKPENRDFLKRVKAEVDS
VLGLRDFITLDDVKQMPLVRYSLVEALRLYPAPPMLIRRCLKEDHLTGVGPFSAGMTIKPGQDVMLATWSLNRDQR
LWGPDADKYNPLRFYTAVHGSPEYKAAGWAGFDPARVRGLYPDENAADFGFIPFGGGGRKCMGDQFAILESSVLL
SMLLRDFSFEAADTVTLGMGATIFAKEGLMMKVTARPPQPPDQDEASPVAVASDALLSSVA*

pTL96: M182-K117-A127At-CYP97H1

MAIGVTAQEYVTHYIQNVAQFFVPMKWEDNIPVVKGSIQAVRNEAVFILLHRYMALSPIYKLCIGPRSVVVISDAVA
VKHILRSEVGKYDKGILAEVLKPIMGKGLIPADTITWLTRRRQLKPAFHQKWLHDQLTLYSTVGNRLVAFLAARPGQ
TIDMQERFCSASLDIIGKAVFNYEFGSITRESPVIQAVYAVMREAERRASSIVPYWQLPGGTREFDQHMKVLDDVLTS
LVEQCVQQVSTEEDEEPQKGNNSLLRFLVEARGQDVTNQQLRDDLMTMLIAGHETTAATLTWALHELTKPENRDF
LKRVKAEVDSVLGLRDFITLDDVKQMPLVRYSLVEALRLYPAPPMLIRRCLKEDHLTGVGPFSAGMTIKPGQDVMLA
TWSLNRDQRLWGPDADKYNPLRFYTAVHGSPEYKAAGWAGFDPARVRGLYPDENAADFGFIPFGGGGRKCMGD
QFAILESSVLLSMLLRDFSFEAADTVTLGMGATIFAKEGLMMKVTARPPQPPDQDEASPVAVASDALLSSVA*

**pTL98: MBP-CYP97A3At**
MKIEEGKLVIWINGDKGYNGLAEVGKKFEKDTGIKVTVEHPDKLEEKFPQVAATGDGPDIIFWAHDRFGGYAQSGL<br>LAEITPDKAFQDKLYPFTWDAVRYNGKLIAYPIAVEALSLIYNKDLLPNPPKTWEEIPALDKELKAKGKSALMFNLQ<br>EPYFTWPLIAADGGYAFKYENGKYDIKDVGVDNAGAKAGLTFLVDLIKNKHMNADTDYSIAEAAFNKGETAMTIN<br>GPWAWSNIDTSKVNYGVTVLPTFKGQPSKPFVGVLSAGINAASPNKELAKEFLENYLLTDEGLEAVNKDKPLGAVA<br>LKSYEEELAKDPRIAATMENAQKGEIMPNIPQMSAFWYAVRTAVINAASGRQTVDEALKDAQTNSSSHHHHHHA<br>NSENLYFQGSFPSTVKNGLSKIGIPSNVLDFMFDWTGSDQDYPKVPEAKGSIQAVRNEAFFIPLYELFLTYGGIFRLTF<br>GPKSFLIVSDPSIAKHILKDNAKAYSKGILAEILDFVMGKGLIPADGEIWRRRRRAIVPALHQKYVAAMISLFGEASDR<br>LCQKLDAAALKGEEVEMESLFSRLTLDIIGKAVFNYDFDSLTNDTGVIEAVYTVLREAEDRSVSPIPVWDIPIWKDISP<br>RQRKVATSLKLINDTLDDLIATCKRMVEEEELQFHEEYMNERDPSILHFLLASGDDVSSKQLRDDLMTMLIAGHETS<br>AAVLTWTFYLLTTEPSVVAKLQEEVDSVIGDRFPTIQDMKKLKYTTRVMNESLRLYPQPPVLIRRSIDNDILGEYPIKR<br>GEDIFISVWNLHRSPLHWDDAEKFNPERWPLDGPNPNETNQNFSYLPFGGGPRKCIGDMFASFENVVAIAMLIRRF<br>NFQIAPGAPPVKMTTGATIHTTEGLKLTVTKRTKPLDIPSVPILPMDTSRDEVSSALS*

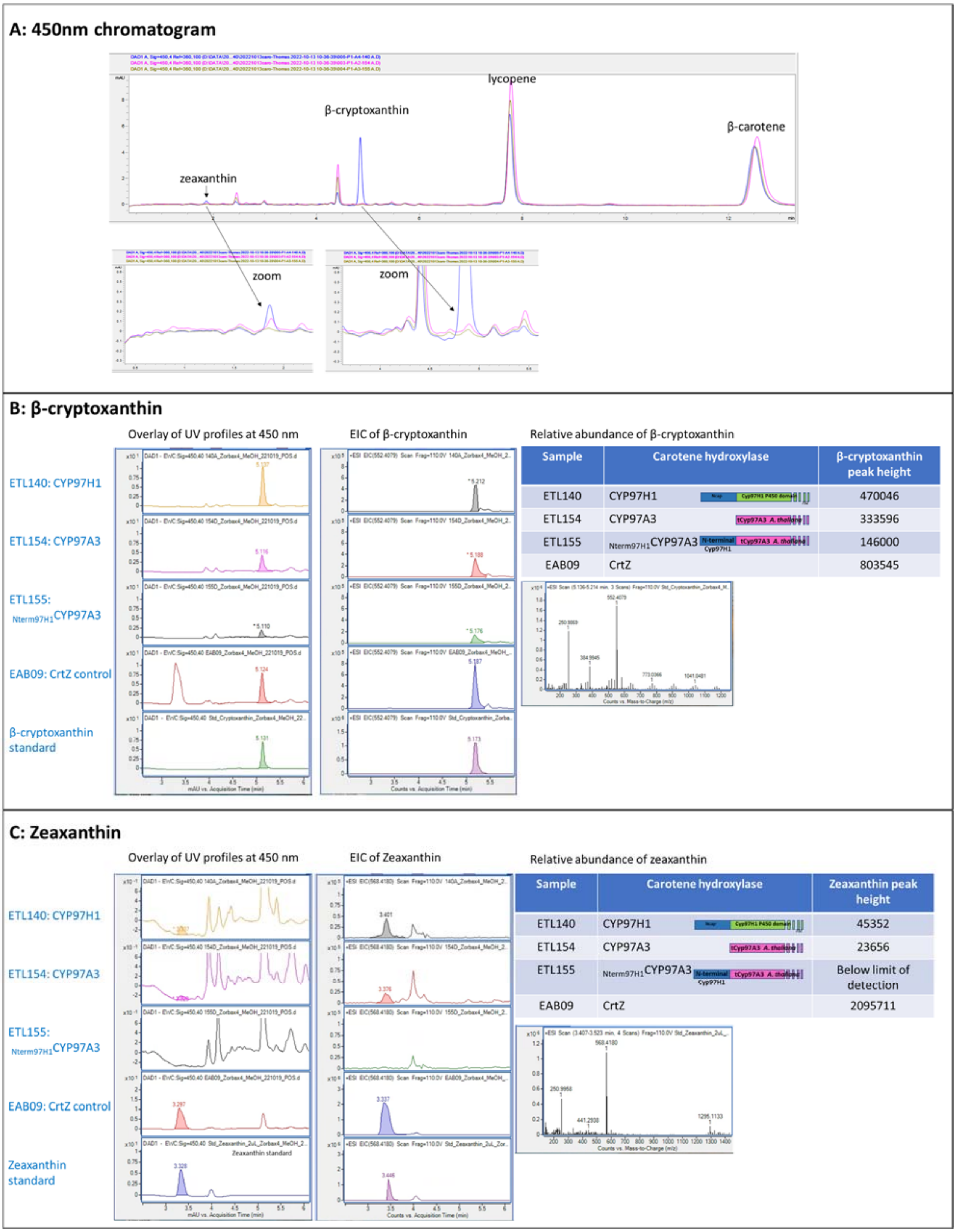

Figure S1. A: full 450nm chromatogram overlay of the acetone extracts from the strains ETL140: CYP97H1 (blue), ETL154: CYP97A3At (pink), ETL155: NtermCYP97H1 fused to CYP97A3At (gold), peaks at 2.4; 2.9 and 4.4 min are also present in the control strain ETL91 which does not express any hydroxylases. **B:** β-cryptoxanthin detection (visible spectra and mass spectrometry analysis). Same samples as in panel A, with the addition of EAB09 (strain expressing the non heme dihydroxylase CrtZ as zeaxanthin producer control). **C:** Zeaxanthin detection (visible spectra and mass spectrometry analysis). Same samples as in panel B.
